# A population-based study of preeclampsia and eclampsia in Ecuador: ethnic, geographical and altitudes differences

**DOI:** 10.1186/s12884-021-03602-1

**Published:** 2021-02-09

**Authors:** Eduardo Tejera, Maria Eugenia Sánchez, Aquiles R. Henríquez-Trujillo, Yunierkis Pérez-Castillo, Marco Coral-Almeida

**Affiliations:** 1 Facultad de Ingeniería y Ciencias Aplicadas, Universidad de Las Américas, Quito, Ecuador; 2 Facultad de Ciencias de la Salud, Universidad de Las Américas, Quito, Ecuador; 3 Escuela de ciencias Físicas y Matemáticas, Universidad de Las Américas, Quito, Ecuador; 4 One Health Research Group, Universidad de Las Américas, Quito, Ecuador

**Keywords:** Ethnic groups, Montubios, Altitude, Preeclampsia, Eclampsia

## Abstract

**Background:**

In Ecuador eclampsia and preeclampsia were identified as the third cause of maternal death. Like other Latin-American countries, Ecuador has human settlements living from 0 to more than 4000 m of altitude and comprising a wide ethnic-diversity across all these altitude changes. These characteristics offer the possibility to study a wide variety of possible risk factors for preeclampsia and eclampsia.

**Methods:**

We conducted a population-based retrospective study of all deliveries in Ecuador from 2015 through 2017. The main variables analyzed were: altitude, ethnic self-identification, geographic location, and maternal age. The data comes from the Ecuadorian National Institute of Statistics and Census (INEC) and the Ecuadorian Ministry of Health. Data information regarding maternal parity and socioeconomic status was not available from official records. Logistic regression analysis was used to study the relationship between preeclampsia and eclampsia with the variable of interest. Geospatial statistical analysis was done to identify statistically significant spatial clusters of preeclampsia and eclampsia cases.

**Results:**

The incidence of preeclampsia was estimated between 5.11 (5.05–5.18) and 6.23 (6.16–6.30), and 0.25 (0.23–0.26) for eclampsia. Native American have a lower incidence regarding preeclampsia compared to other ethnic groups. High altitude has a significant odds ratio (OR = 2.31, 1.93–2.78) of preeclampsia. Montubio residing in middle altitude (1500–3500 m) have the highest risk of preeclampsia (OR = 18.13, 9.53–34.50). Afro-Ecuadorians also have an increased risk of preeclampsia associated with altitude (OR = 2.36, 1.78–3.14). Ethnicity was not identified as a risk factor for eclampsia. Early and older maternal age was associated with an increased risk of preeclampsia and eclampsia. Women living more than 20 km from the obstetric unit have an OR = 2.61 (2.32–2.95, *p*-value< 0.01) and OR = 1.87 (1.82–1.92, p-value< 0.01) of developing eclampsia and preeclampsia respectively.

**Conclusions:**

Preeclampsia is widespread across low and high-altitude areas, while eclampsia is mostly located at lower altitudes. Montubios living at middle or high altitudes represents the ethnic group with a higher risk of preeclampsia. No ethnic effect was identified as a potential risk factor for eclampsia. Moreover, in eclampsia the associated risk of young women seems to be higher than in preeclampsia.

**Supplementary Information:**

The online version contains supplementary material available at 10.1186/s12884-021-03602-1.

## Background

Hypertensive disorders during pregnancy are a major cause of maternal and fetal mortality and morbidity worldwide [1]. The major factors associated with preeclampsia are maternal weight, age and as well as personal and family history of diabetes and hypertension [2, 3]. An increased incidence in Latin American countries was previously reported and related to several factors like socioeconomic status, country developments and improvement in diagnosis [4, 5]. Late care-seeking behavior and diagnosis and management delay are important risk factors for increased morbidity and mortality among women with preeclampsia [6]. A recent study found that nulliparous women living more than 1 h from obstetric units have a 50% increased risk of eclampsia [6]. Previous works had also pointed toward the influence of high altitude in pregnancy outcome specially preeclampsia [7–9]. Like other Latin-American countries, Ecuador, has human communities living from 0 to more than 4000 m above sea level (m.a.s.l.) comprising a wide ethnic-diversity across all these altitude changes. Together with the high number of deliveries in women under 18 years old, these characteristics bring the possibility to study a wide variety of effects during some pregnancy-related diseases.

In Ecuador, according to the National Institute of Statistics and Census (INEC), eclampsia and preeclampsia were identified as the third cause of maternal death after preexisted chronic diseases and postpartum hemorrhage [10]. This study aimed to describe the epidemiology of preeclampsia and eclampsia in Ecuador, and the influence of demographic factors, geographical location, altitude, and ethnic background. While some similar studies had been reported previously in other Latin-American countries like Peru [5, 11–13], Chile [5], Brazil [5, 14, 15] and Argentina [5, 16, 17], in Ecuador, this is the first epidemiological study covering these aspects.

## Methods

### Study design

We conducted a population-based retrospective study including all deliveries registered in Ecuador from 2015 through 2017. The main variables under study were: altitude, ethnic self-identification, geographic location and maternal age.

### Dataset

The data used in this study comes from the national registries of hospital discharges published by the Ecuadorian National Institute of Statistics and Census (INEC) and the Ecuadorian Ministry of Health (MoH) for the period 2015–2017 [18]. All cases were identified and grouped by their three-character ICD-10 code: O14 for preeclampsia and O15 for eclampsia. The database comprises all hospitalized individuals and for this study, only Ecuadorian women with subsequent delivery were considered. All records are anonymous and comprise maternal age, residence location, hospital location, hospitalization date, clinical diagnosis, ethnic self-identification and delivery type. The demographic distribution is presented in Table 1.

**Table 1:** Demographic characteristics of the population

	2015	2016	2017
**Maternal age (years) (%)**			
10–14	1584 (0.94)	1430 (0.90)	1427 (0.85)
15–19	35,596 (21.2)	31,121 (19.5)	31,756 (19.0)
20–24	44,549 (26.6)	41,535 (26.1)	43,886 (26.3)
25–29	38,051 (22.7)	36,861 (23.1)	39,380 (23.6)
30–34	28,308 (16.9)	28,395 (17.8)	30,071 (18.0)
35–39	14,902 (8.9	15,382 (9.7)	15,922 (9.5)
40–44	4231 (2.5)	4259 (2.7)	4096 (2.5)
45–49	429 (0.26)	440 (0.28)	395 (0.24)
50–55	0 (0.00)	12 (0.01)	27 (0.24)
**Deliveries**	167,650	159,435	166,960
**Ethnicity (%)**			
Native American	7296 (4.35)	7179 (4.50)	7261 (4.35)
Afro Ecuadorian	2622 (1.56)	1963 (1.23)	1918 (1.15)
Montubio	333 (0.20)	380 (0.24)	633 (0.38)
Mestizo	144,569 (86.2)	140,634 (88.2)	146,213 (87.6)
Caucasian	721 (0.43)	483 (0.30)	527 (0.32)
Other/Non-Specified	12,109 (7.2)	8796 (5.5)	10,408 (6.2)
**Altitude (m) (%)**			
< 1500	98,777 (58.9)	89,137 (55.9)	101,486 (60.8)
1500–3500	56,875 (33.9)	57,162 (35.9)	52,604 (31.5)
> 3500	11,627 (6.9)	12,904 (8.1)	12,574 (7.5)
Non-Specified	371 (0.22)	232 (0.15)	296 (0.18)

Note: Percentage is indicated in parenthesis

The INEC classifies ethnic self-denomination as: Native Americans, Afro Ecuadorians, Blacks, Mulatos, Montubios, Mestizos, Caucasians, Others and Ignored. In this study Afro Ecuadorians, Blacks and Mulatos were grouped as “Afro Ecuadorian” (Table 1) while the categories “ignored” and “others” were excluded from the ethnicity analysis. Montubio population is a specific self-defined ethnic mestizo sub-group geographically located in rural areas on the Ecuadorian coast, and most of them work as peasants [19]. We decided to include them as a separated group and not as part of the Native American population because they are delimited in a well-defined geographical location and have their own cultural and phenotypic characteristics distinct from those of the other Native American population in Ecuador.

The hypertensive disorders are classified in the database as non-specified preeclampsia, moderate and severe preeclampsia, gestational hypertension without proteinuria, gestational hypertension with proteinuria, eclampsia (EC), HELLP syndrome, and non-specified gestational hypertension. We grouped as preeclampsia (PEC) the following diagnosis: non-specified preeclampsia, gestational hypertension without proteinuria, gestational hypertension with proteinuria, moderate and severe preeclampsia. Non-specified gestational hypertension was not considered. In 2013, the American College of Obstetricians and Gynecologists and similarly The International Society for the Study of Hypertension in Pregnancy (ISSHP) elected to exclude proteinuria as a necessary condition to be met to establish the clinical diagnosis of preeclampsia in the presence of other maternal organ dysfunction or uteroplacental dysfunction [20]. Based on this consideration and to reduce the risk of possible misclassification in the national records we decided to include “gestational hypertension without proteinuria” in the same categories. However, to discard any possible effect because of this consideration in incidence and factors analysis, we will present both results in all tables: preeclampsia including all cases and preeclampsia excluding all cases of gestational hypertension without proteinuria. Altitude was obtained by using the patient’s residence address and categorized into tree groups: altitude inferior to 1500, between 1500 and 3500 and higher than 3500 m above sea level (m.a.s.l.). Maternal age was categorized as follows: 10–14, 15–19, 20–24, 25–29, 30–34, 35–39, 40–44 and 45–49 years old.

### Statistical analysis

Logistic regression analysis was used to study the relationship between the frequency of PEC or EC cases and demographic variables such as ethnicity, maternal age, the altitude of the place of residence and distance from residence to the obstetric health care facility. The estimated effects are expressed as odds ratio (OR) and 95% confidence intervals (CI). We also used step-backward and step-forward analysis using the Wald method for model selection. In these strategies for stepwise selection the entries are tested based on the significance of the score statistic, and variable removal is tested using the probability of the Wald statistic. Wald statistic or Wald Chi-Squared test is used to evaluate the explanatory variable’s relevance for its exclusion (or not) from the model. Maternal age was not treated as a categorical variable in logistic regression analysis but as a continuous variable. For the study of the altitude and ethnicity, the relative risk was computed using binary logistic regression for PEC and EC. We included the maternal age (MA) and its quadratic transformation (MA^2^) to explore nonlinear behavior if present. PEC and EC incidences were calculated for the total number of normal deliveries (natural and cesarean) using the Poisson distribution for confidence interval estimation using the “stats*”* R-package. The geographical distance was calculated using the GeoPy Python library [21] and transformed to its natural logarithm. The distance was computed from GPS coordinates from each canton (second sub-division of Ecuador after the provinces) provided by the INEC.

### Geospatial statistical analysis

The spatial units for the analysis were the cantons. In Ecuador, Cantons are second-order territorial and administrative subdivisions smaller than the provinces and governed by a municipality and its respective mayor. Each canton may have several parishes, both urban or rural.

All EC and PEC cases were handled as new cases in order to obtain relative risk estimates. Geospatial statistical analysis was done to identify statistically significant spatial clusters of PEC and EC cases. The spatial clusters were defined by the cantons with higher incidence of EC and PEC cases. Each EC and PEC cases were distributed geographically by canton. The spatial analysis was done in SATSCAN v9.6 [22] while visualization of the special analysis was conducted using QGIS version 3.8 Zanzibar software [23] . Shape files used for all the maps in this article were obtained from the INEC portal following their licensing requirements [18]. All maps were created and designed by the authors of this manuscript.

For the geospatial analysis we used three types of information: 1) the number of eclampsia and preeclampsia cases distributed in each canton, 2) the total population in each canton (which in this case is the total number of deliveries) and c) the geographical coordinates of each canton. The Poisson distribution was used to compare the count of eclampsia and preeclampsia cases in each canton. Space clustering was assessed by comparing the incidence rate ratio of the EC and PEC cases within a specific geographical area in contrast to an expected incidence rate ratio of EC and PEC cases if their incidences were randomly distributed. A likelihood ratio test was used to check the significance of identified space clusters and Monte Carlo simulations (999 runs) were used to obtain the *p*-values of the test. A cluster was identified as significant if the p-values were inferior to 0.05 [24]. Additionally, the Gini coefficient was used as a selection filter amongst the significant clusters [25]. The same methodology was also used to identify the spatial clusters of deliveries for each specific ethnic group.

## Results

The incidence of preeclampsia (PEC) and eclampsia (EC) is presented in Table 2.

**Table 2:** Preeclampsia and eclampsia incidence for our entire cohort

Prevalence (%)	2015 (CI:95%)	2016 (CI:95%)	2017 (CI:95%)	Overall (CI:95%)
Preeclampsia	5.18 (5.08–5.29)	6.57 (6.45–6.70)	6.95 (6.82–7.08)	6.23 (6.16–6.30)
Preeclampsia^1^	4.07 (3.97–4.17)	5.38 (5.26–5.49)	5.91 (5.80–6.29)	5.11 (5.05–5.18)
Eclampsia	0.20 (0.18–0.23)	0.24 (0.22–0.26)	0.29 (0.27–0.32)	0.25 (0.23–0.26)

Note: 1) Incidence and confidence interval were computed, excluding all cases of gestational hypertension without proteinuria. Intervals are presented as confidence intervals at 95% (CI:95%)

The number of preeclampsia cases increased from 8961 in 2015 to 11,609 in 2017 (a total of 15,390 across all years). In the same period, cases of gestational hypertension without proteinuria (GHWP) decreased from 1870 in 2015 to 1739 in 2017 (a total of 5519 across all years). In eclampsia, we also noticed a modest increment from 343 cases in 2015 to 488 cases in 2017 (a total of 1214 across all years).

**Fig. 1:**
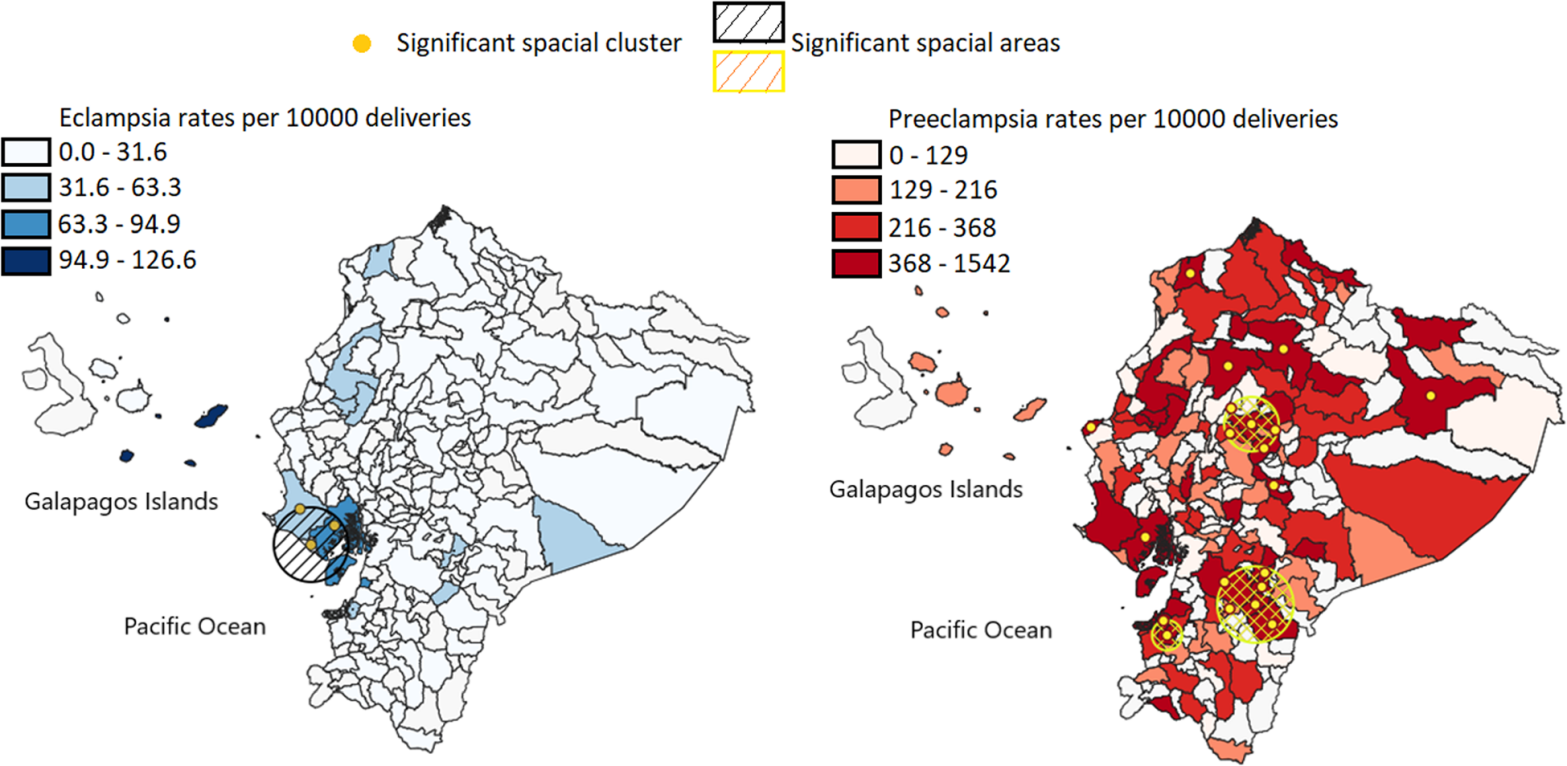
Geographical clusters indicating the areas with incidence of eclampsia (Left) and preeclampsia (Right). The maps were created by the authors as described in the methodology

Moreover, the geographic distribution pattern of both diseases is different across the country (Fig. 1). Preeclampsia is more widespread than eclampsia, including low and high-altitude areas, while eclampsia is mainly located at a lower altitude (a map with altitude ranges of Ecuador is presented in supplementary material 1). The relative risk ratio in the cluster for eclampsia was of 3.96 while for preeclampsia, it varied from 1.19 to 2.56 compared to areas outside the clusters.

### Effect of maternal age, altitude and ethnicity in preeclampsia

We can notice from Table 3 that the odd ratios computed for altitude, ethnicity, maternal age and geographical distance do not differ too much by removing all cases of gestational hypertension cases without proteinuria (GHWP). Native Americans and Montubio had a lower propensity regarding preeclampsia than other ethnic groups. However, the altitude effect is quite different (Table 3). We can notice a statistically significant increased risk (OR = 2.31) over the 3500 m of altitude.

**Table 3:** Influence of ethnicity, altitude and maternal age in preeclampsia incidence and also excluding all gestational hypertension cases without proteinuria

Variables			***p***-value	OR	95% CI OR		***p***-value^**1**^	OR1^**1**^	95% CI OR^**1**^	
Ethnicity^a^	Afro Ecuadorian		< 0.001	2.34	1.95	2.81	< 0.001	2.18	1.79	2.66
	Montubio		0.001	0.46	0.29	0.73	0.001	0.42	0.25	0.70
	Mestizo		< 0.001	1.80	1.54	2.10	< 0.001	1.75	1.49	2.07
	Caucasians		0.311	1.17	0.87	1.58	0.276	1.20	0.87	1.66
Altitude^b^	1500–3500		0.004	0.76	0.63	0.92	0.003	0.73	0.60	0.90
	> 3500		< 0.001	2.31	1.93	2.78	< 0.001	2.40	1.97	2.93
Altitude x Ethnicity^c^	1500–3500	Afro Ecuadorian	< 0.001	2.36	1.78	3.14	< 0.001	2.86	2.11	3.89
	1500–3500	Montubio	< 0.001	18.13	9.53	34.50	< 0.001	22.32	11.02	45.22
	1500–3500	Mestizo	0.211	1.13	0.93	1.36	0.052	1.23	1.00	1.505
	1500–3500	Caucasians	< 0.001	2.95	1.81	4.80	< 0.001	3.06	1.81	5.20
	> 3500	Afro Ecuadorian	0.170	0.59	0.28	1.25	0.035	0.32	0.11	0.92
	> 3500	Montubio	0.093	4.06	0.79	20.8	0.052	5.17	0.99	27.00
	> 3500	Mestizo	< 0.001	0.54	0.45	0.65	< 0.001	0.56	0.46	0.69
	> 3500	Caucasians	0.285	1.66	0.65	4.23	0.700	1.24	0.43	3.73
Maternal Age	MA		< 0.001	0.92	0.91	0.93	< 0.001	0.91	0.90	0.92
	MA^2^		< 0.001	1.00	1.00	1.00	< 0.001	1.00	1.00	1.00
Geographical Distance			< 0.001	1.18	1.17	1.18	< 0.001	1.20	1.19	1.21

Notes: 1) The “1” super index for OR, confidence interval and *p*-values were computed excluding all gestational hypertension cases without proteinuria. (^a^) In these variables the Native American were considered as reference. (^b^) In altitude the group of women living < 1500 mts over the sea level were considered as reference. (^c^). In this interaction the Native American leaving at < 1500 mts over sea level was considered as the reference group. The maternal age (MA) was included also in the quadratic form (MA^2^). All computation was performed including all variables in the model: ethnicity, altitude and maternal age

In terms of interaction analysis, we can notice some variations if we remove all cases with GHWP. However, a closer analysis indicates that major variations are actually in the cases were the *p*-value is higher than 0.05, specifically the odd ratios of Montubios and Afro Ecuadorians at high altitudes that we will discuss later.

**Fig. 2:**
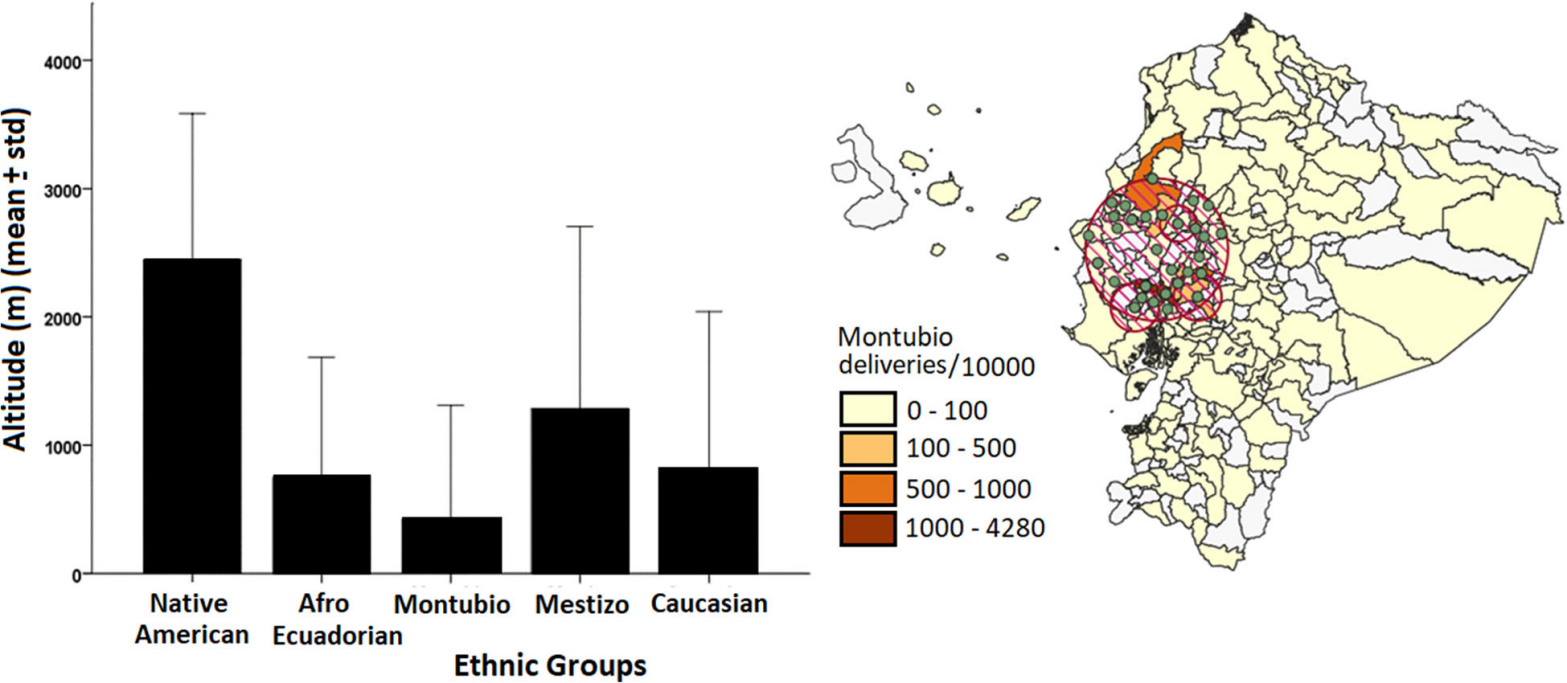
Left) Average altitude variation with respect to ethnic groups. Right) Geographical clusters indicating the areas with major location of Montubios community. The maps were created by the authors as described in the methodology

The cross-altitude variation of Native American and the fact that they are probably better adapted to those different altitudes was the main reason for choosing them as the reference category in logistic regression (Table 3, Fig. 2, Left). The interaction terms in logistic models bring more light about the effect of altitude and ethnicity. Montubio communities residing in altitude have the highest risk (OR = 18.13, 9.53–34.50). In general, Montubios communities and Afro-Ecuadorian communities are located at lower altitudes (Fig. 2, Right) while Native American, and even mestizos, is located across a wide spectrum of altitude (Fig. 2, Left). However, those who move to middle altitudes (1500–3500 m, in which the capital Quito is located) or high altitudes (> 3500 m) clearly shows an elevated risk of preeclampsia development. Even if the odds ratio of Montubios at higher altitudes remains statistically significant after removing all cases of GHWP, we should keep in mind that the Montubio community is actually very small (Table 1). In middle altitude, we have a total of 133 deliveries in the Montubio community including 25 cases of preeclampsia. However, at a lower altitude we have 441 deliveries, including 21 cases of preeclampsia. It is easy to notice a considerable increment of cases in middle altitude. At altitudes above 3500 m, only 11 deliveries were reported with Montubios ethnic self-identification. In fact, at high altitude the most abundant communities are Native Americans (*n* = 4744, altitude > 3500 m) and Mestizo (*n* = 28,718, altitude > 3500 m).

**Fig. 3:**
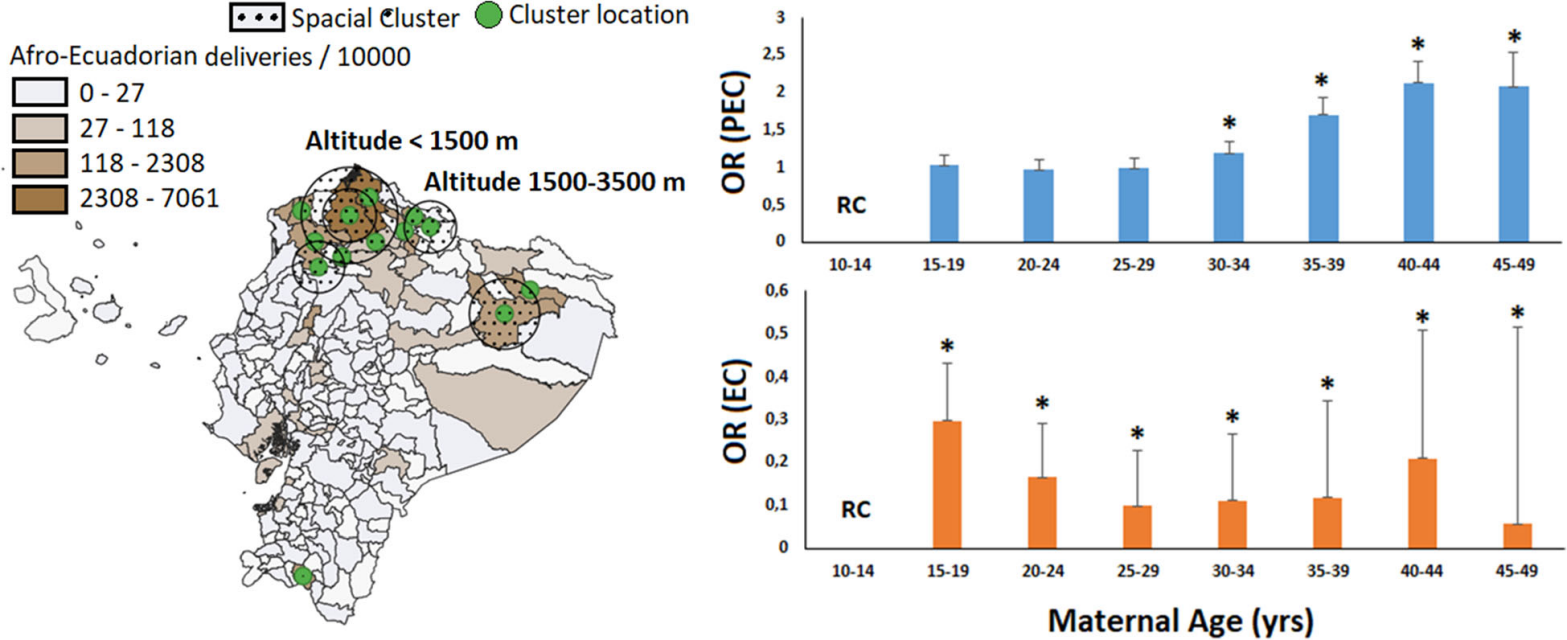
Left) Significant population cluster of Afro-Ecuadorian and the associated altitude. Right) Variation of the adjusted odd ratio in each maternal age group. The notation “RC” indicates the reference class which correspond to youngest women and (*) correspond with p-values less than 0.01 with respect to the reference class. The maps were created by the authors as described in the methodology

The Afro-Ecuadorian community has an increased risk compared with Caucasians and Native American communities (OR = 2.34, 1.95–2.81) and associated with altitude. Moreover, we should notice that no significant Afro Ecuadorian community lives higher than 3500 m (Fig. 3, Right). At higher altitude over 3500 m only 65 deliveries were reported with Afro-Ecuadorian identification, nine reported with preeclampsia (and decreases to four if we remove cases with GHWP). This reduced number does not support of a protective effect, as suggested in Table 2 with *p*-value = 0.035. More cases need to be considered in order to corroborate this result. The two major Afro Ecuadorian communities are located at low and middle altitude (Fig. 3, Right). According to our results, the prevalence is higher between 1500 and 3500 m. The farthest Afro-Ecuadorian community (Fig. 3, Right) correspond with the Amazonian area. In middle altitude, we have a total of 952 deliveries in the Afro-Ecuadorian community, from which 123 developed preeclampsia (111 cases by removing GHWP cases).

The statistical significance regarding maternal age (including quadratic term MA^2^) confirm maternal age’s quadratic behavior. To have a clear presentation, we used the categorized age as in Table 1 to calculate the adjusted risks (Fig. 3, Left). Our results confirm that maternal age over 35 years old is associated with an increment in preeclampsia risk.

### Effect of maternal age, altitude and ethnicity in eclampsia

The ethnicity and altitude have very different behavior in eclampsia regarding preeclampsia. No difference in ethnicity was found and the altitude has an inverse effect. Higher altitude has a protective effect on eclampsia (Table 4). The total number of cases with eclampsia is low (*n* = 1214). The distribution of cases across altitude is 14, 21 and 12 in < 1500 m, 1500–3500 m and > 3500 m respectively. Even when there are small numbers, they are similar across all altitudes. However, the distribution of these cases across such heterogeneous data as ethnicity will reduce the number of cases considerably limiting the statistical analysis.

**Table 4:** Influence of ethnicity, altitude and maternal age in eclampsia prevalence

Variables		***p***-value	OR	95% CI OR	
Altitude^a^	1500–3500	< 0.001	0.64	0.55	0.74
	> 3500	0.007	0.69	0.54	0.89
Maternal Age	MA	< 0.001	0.69	0.65	0.72
	MA^2^	< 0.001	1.01	1.00	1.01
Geographical Distance		< 0.001	1.30	1.26	1.33

Notes: (^a^) In altitude the group of women living < 1500 mts over the sea level were considered as reference. The maternal age (MA) was included also in the quadratic form (MA^2^). All computation was performed including all variables in the model but were not significant as well as interactions

Similar to preeclampsia, we also confirm the quadratic behavior of maternal age for eclampsia incidence. However contrary to preeclampsia, extremes ages, especially younger women (Fig. 3, Left) have the highest risk of developing eclampsia than other ages. From all cases of preeclampsia (*n* = 15,390) 250 (1.62%) were reported in women with age between 10 and 14 years old, while from all cases of eclampsia (n = 1214) 68 (5.60%) were reported in the same age interval.

**Fig. 4:**
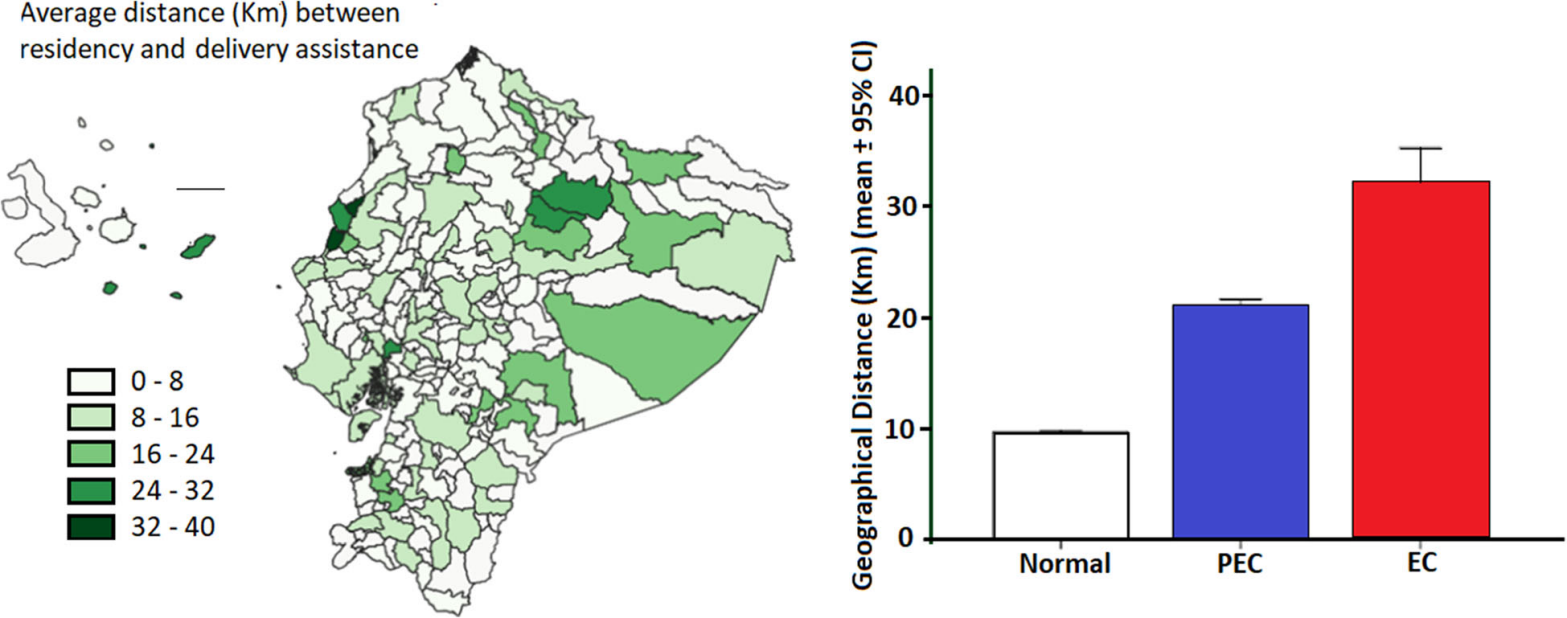
Left) Canton distribution of the average distance between residency and partum assistance. Right) Average distribution of geographical distance between the location of residence and obstetric unit with respect to diseases conditions. The maps were created by the authors as described in the methodology

We can notice (Fig. 1 Left) that some Galapagos Islands (San Cristobal, Española and Floreana) apparently have a higher relative risk mainly for eclampsia. However, we also noticed that some of the same factors also shown high geographical distance between the residence and attention locations (Fig. 4 Right). In Table 3 and Table 4 the geographical distance between residence and attention units are significantly related to incidences and with OR higher than 1. After categorizing distance for 20 km between residence and health care facility we found an OR = 2.61 (2.32–2.95) for eclampsia and OR = 1.87 (1.82–1.92) for preeclampsia. This calculation includes all the other factors (maternal age, altitude, and ethnicity).

## Discussion

Parallel to the overall increment in preeclampsia cases, we noticed a small reduction in gestational hypertension without proteinuria. This small reduction suggests that possibly some of these cases (at least in the first years) can be associated with preeclampsia and not necessarily with gestational hypertension. We think that the diagnosis was improved through 2017. However, even after all gestational hypertension cases without proteinuria, we see a small increment in preeclampsia incidence from 2015 to 2017. The increment over this period can be associated with improvement in clinical diagnosis as well as database annotation. The incidence obtained, which should be between 5.11 (CI 5.05–5.18) and 6.23 (CI 6.16–6.30) is higher than other values reported in US [3, 26], Australia [27] but similar to those found in Argentina, Mexico, Chile [5] and even Brazil [14]. These differences could be associated with several socio-economic factors and gestational age at delivery or even the interval between gestations [4, 14].

Initially we decide to include all cases of GHWP in the preeclampsia group because possibly some of these cases were actually preeclampsia and not gestational hypertension. Our data suggest that our initial consideration was not entirely wrong. We noticed that in the logistic model the interacting terms but no independent terms seem to be partially affected by excluding all cases of GHWP. The confidence intervals of the odd ratio values obtained in independent terms shown considerable superposition between both datasets.

The effect of high altitude on reducing fetal weight had been previously identified [11, 16]. Moreover, for preeclampsia epidemiological and molecular studies also support an increased risk regarding high altitude [7, 9]. However, genetic adaptation to hypoxia conditions could act as a protective factor regarding preeclampsia and other pathologies [8]. We should expect to identify this adaptation or “inadaptation” by ethnic and geographical analysis in our dataset. We can notice that the Montubio community residing in altitude have the highest risk. This effect was not detected in the general variable because the majority of Montubios live close to the coast. Contrary to general Native American community who lives almost across the entire country, the Montubios are historically located closer to the coast and consequently at low altitudes. However, those who move to middle (1500–3500 m, in which the capital Quito is located) or high altitude (> 3500 m) clearly shown an elevated risk of preeclampsia development. This could be an example of a relationship between genetic background and altitude adaptability. Even when the odds ratio of Montubios at middle altitude remains statistically significant after removing all cases of GHWP, we should keep in mind that the Montubio community is actually very small (Table 1). This sample is even more reduced at high altitudes and it is probably the main reason behind no significant interaction.

Concerning the Afro Ecuadorian community, we confirm what other authors already found in other countries. Afro Ecuadorian communities have an increased risk compare with Caucasians and Native American communities and are also associated with altitude [5, 26]. We can notice that over the 3500 m we did not detect statistical differences (even when the confidence interval is quite wide) and the apparent significance regarding removing all cases with GHWP is based on the reduced Afro-Ecuadorian population size at that altitude. The main reason behind this pattern is that any significant Afro Ecuadorian community live higher than 3500 m. The two major Afro Ecuadorian communities are located at low and middle altitude (Fig. 3 Right). Both communities are geographically constrained and had also been living in the same area for many generations. According to our results the incidence of preeclampsia is higher between 1500 and 3500 m. The farthest community (Fig. 3 Right) correspond with Amazonian area and there is not much information available regarding this zone.

Besides all previous discussion regarding the influence of ethnicity on altitude, we should also consider that higher odds ratios in higher altitudes (over 3500 m) can also be associated with possible socioeconomic status modifications. Important cities in economic terms like Quito (Ecuador capital) and Guayaquil (the second more important economical city) are located at the middle and low altitude. No major cities with strong economic activity are located at high altitude and therefore, is logical to think that on those areas we should consider possible factors like modifications of the socioeconomic as well as the possible implication in early diagnosis and management of these hypertensive diseases.

The statistical significance regarding maternal age confirm the quadratic behavior (MA and MA^2^) concerning preeclampsia and eclampsia incidence (Tables 3 and 4 and Fig. 3 Left). The Fig. 3 (Left) confirm that increased maternal ages are associated with an increment in preeclampsia risk. The influence of increased maternal age upon preeclampsia risk is a well-known relationship, but the risk associated with young ages is controversial. Previous authors pointed out that extreme ages have an increased risk regarding preeclampsia prevalence [3, 26]. But while young age tends to increase the risk of late-onset, older ages are a risk factor for early-onset preeclampsia [26]. From our data, it was not possible to differentiate early-onset from late-onset preeclampsia but the quadratic pattern is statistically significant. However, a very different pattern was found concerning the influence of maternal age and eclampsia. The extreme maternal ages but especially younger women have an increased risk of developing eclampsia. As presented, the crude percentage of all eclampsia cases in the 10–14 years old interval is almost 5 times higher than preeclampsia for the same age interval. Several non-biological factors can be responsible for these patterns including child abuse and socio-economic status. Unfortunately, information regarding socioeconomic status of the patients in our datasets was not available. Younger women suffering from child abuse could not be subjected to periodic antenatal visits and therefore progress toward complications only noticed at late pregnancy states.

While preeclampsia can be identified at antenatal visits, some women experience a sudden and rapid progression to eclampsia or even HELLP syndrome [6, 28]. Moreover, late care seeking and diagnosis and management delays are important risk factors for increased morbidity and mortality among women with preeclampsia [6]. A recent study found that nulliparous women living more than 1 h form obstetric units have a 50% increased risk of eclampsia [6]. Considering the heterogeneous geographical topology in Ecuador, we also analyzed the effect of distance between residence and obstetric attention unit in preeclampsia and eclampsia incidence. We can easily notice that the distance between residence and obstetric attention units is higher in eclampsia compared to preeclampsia and in general compared to normal pregnancy (Fig. 4. Right). Geographical distance to the attention center was largest for eclampsia cases. The relative risk of eclampsia associated with geographical distance beyond 20 Km is higher than the value obtained for preeclampsia adjusting for the same variables. The increment of the eclampsia risk associated with geographical distance may not only be associated with rapid progression but also with: 1) severe obstetric complications may be transferred to specialized hospital usually placed on non-rural areas, 2) in rural areas women are less enrolled in periodic antenatal care, which would increase the risk to rapidly progress from undiagnosed PEC to EC.

### Strength and limitations

The public data used does not have information regarding women number of gestation and/or parity and we also ignore if preeclampsia corresponds to late or early onset diagnosis. The data does not discriminate if the presented preeclampsia/eclampsia event was the first event or if it is recurrent. The socioeconomic information is also absent. These variables are relevant to preeclampsia and eclampsia physiopathology, developments and have a direct impact in the presented incidences. The socioeconomic status varied across the entire country an also may vary across ethnic groups.

Knowing that preeclampsia is more likely to happen in primigravid women, this work’s reported values may be higher in this group. A similar implication can be discussed regarding maternal age. We noticed an increment in preeclampsia incidence in very young women. However, it is probably that in this group the socioeconomic status and parity (very likely they are primigravid) play an important role in the reported incidence of preeclampsia and eclampsia. Similarly, in women with advanced maternal age we also noticed an increment in the incidence of preeclampsia and eclampsia. Even when we know that advanced maternal age is a risk factor for preeclampsia, we need to consider that we cannot adjust for parity, possible cases of recurrent preeclampsia or other hypertension problems during previous pregnancy in our reported incidences.

Despite these limitations, this is the first population-based study in Ecuador. The study comprises information from the entire country and could provide guides for the Public Health management and future studies in other fields like population genetic and biomarkers discovery.

## Conclusions

Preeclampsia is widespread across low and high-altitude areas while eclampsia is mainly located at lower altitude. Ethnicity and altitude shown a significant effect on the risk of preeclampsia but not eclampsia. Caucasian and Afro-Ecuadorian have an increased risk of developing preeclampsia regarding altitude. However, Montubios living at middle or high altitude represent the ethnic group with higher risk of preeclampsia.

We found that the youngest and the oldest age group have an increased risk of developing both preeclampsia and eclampsia. However, in eclampsia the associated risk of young women seems to be higher than in preeclampsia. We also found that women living more than 20 Km from the hospital also have an increased risk for both hypertensive disorders. Although, the relative risk of eclampsia seems higher than for preeclampsia regarding geographical distance.

## Supplementary Information

**Additional file 1: Fig. 1**. Map of Ecuador indicating altitude distribution by Canton in meter above sea level (MASL). The maps were created by the authors as described in the methodology.

## Data Availability

All the data used in this manuscript is publically available through the Ecuadorian National Institute of Statistics and Census (INEC) and the Ecuadorian Ministry of Health (MoH) at: https://www.ecuadorencifras.gob.ec/camas-y-egresos-hospitalarios/.

## Abbreviations

CI: Confidence intervals
EC: Eclampsia
INEC: Ecuadorian National Institute of Statistics and Census
MA: Maternal age
MA^2^: Quadratic transformation of maternal age
MASL: Meters above the sea level
MoH: Ecuadorian Ministry of Health
OR: Odds ratio
PEC: Preeclampsia
RC: Reference class
GHWP: Gestational hypertension without proteinuria
