# Supplementary material for: A population-based study of preeclampsia and eclampsia in Ecuador: ethnic, geographical and altitudes differences
Source: BMC Pregnancy Childbirth. 2021 Feb 9;21:116. doi: 10.1186/s12884-021-03602-1 (PMC7874663; doi:10.1186/s12884-021-03602-1)
Supplement: Supplementary file 1 — Additional file 1: Fig. 1. Map of Ecuador indicating altitude distribution by Canton in meter above sea level (MASL). The maps were created by the authors as described in the methodology. [file 12884_2021_3602_MOESM1_ESM.docx]

# A population based study of preeclampsia and eclampsia in Ecuador: ethnic, geographical and altitudes differences


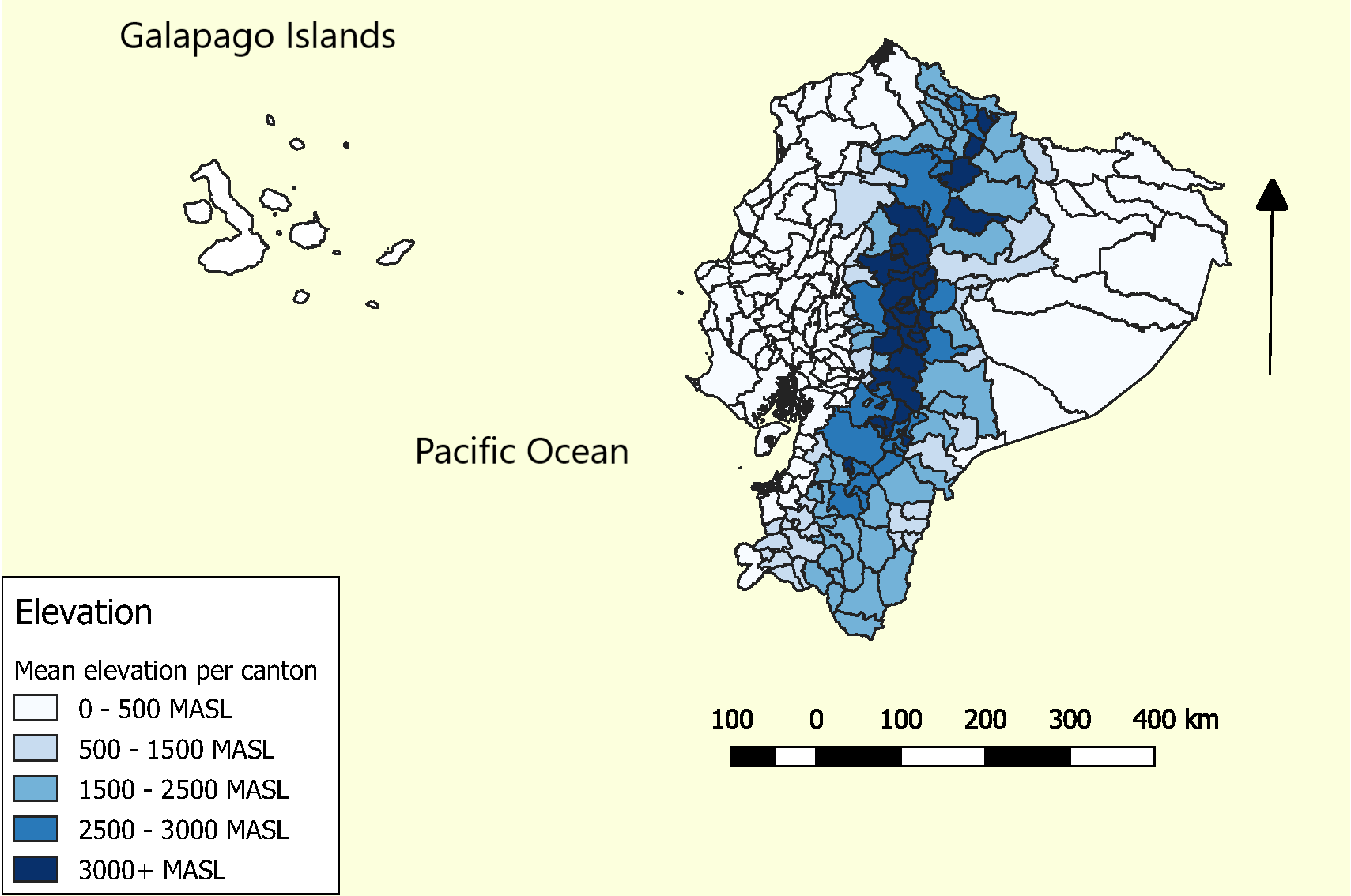


Figure 1. Map of Ecuador indicating altitude distribution by Canton in meter above sea level (MASL). The maps were created by the authors as described in the methodology.
